# Complete chloroplast genome of seven *Fritillaria* species, variable DNA markers identification and phylogenetic relationships within the genus

**DOI:** 10.1371/journal.pone.0194613

**Published:** 2018-03-15

**Authors:** Yan Li, Zhirong Zhang, Junbo Yang, Guanghui Lv

**Affiliations:** 1 Institute of Arid Ecology and Environment, Xinjiang University, Urumqi, Xinjiang, China; 2 Key Laboratory of Oasis Ecology, Xinjiang University, Urumqi, Xinjiang, China; 3 Germplasm Bank of Wild Species in Southwest China, Kunming Institution of Botany, Chinese Academy of Sciences, Kunming, Yunnan, China; National Cheng Kung University, TAIWAN

## Abstract

*Fritillaria* spp. constitute important traditional Chinese medicinal plants. Xinjiang is one of two diversity hotspots in China in which eight *Fritillaria* species occur, two of which are endemic to the region. Furthermore, the phylogenetic relationships of Xinjiang *Fritillaria* species (including *F*. *yuminensis*) within the genus are unclear. In the present study, we sequenced the chloroplast (cp) genomes of seven *Fritillaria* species in Xinjiang using the Illumina HiSeq platform, with the aim of assessing the global structural patterns of the seven cp genomes and identifying highly variable cp DNA sequences. These were compared to previously sequenced *Fritillaria* cp genomes. Phylogenetic analysis was then used to evaluate the relationships of the Xinjiang species and assess the evolution of an undivided stigma. The seven cp genomes ranged from 151,764 to 152,112 bp, presenting a traditional quadripartite structure. The gene order and gene content of the seven cp genomes were identical. A comparison of the 13 cp genomes indicated that the structure is highly conserved. Ten highly divergent regions were identified that could be valuable in phylogenetic and population genetic studies. The phylogenetic relationships of the 13 *Fritillaria* species inferred from the protein-coding genes, large single-copy, small single-copy, and inverted repeat regions were identical and highly resolved. The phylogenetic relationships of the species corresponded with their geographic distribution patterns, in that the north group (consisting of eight species from Xinjiang and Heilongjiang in North China) and the south group (including six species from South China) were basically divided at 40°N. Species with an undivided stigma were not monophyletic, suggesting that this trait might have evolved several times in the genus.

## Introduction

The genus *Fritillaria* L. (Liliaceae) consists of approximately 140 species and is widely distributed in Europe (mostly in the Mediterranean region), Central Asia, China, Japan, and North America [1]. Twenty-four species occur in China, of which 15 are endemic. They are distributed throughout most provinces in China, among which Sichuan and Xinjiang constitute two diversity hotspots. Seven species occur in Xinjiang, and *F*. *tortifolia* X.Z.Duan & X.J.Zheng and *F*. *yuminensis* X.Z.Duan are endemic to this region. Two further species, *F*. *tachengensis* X.Z.Duan & X.J.Zheng (endemic) and *F*. *ferganensis* Losinsk, recorded in Flora *Xinjiangensis*, were reduced to the synonyms of *F*. *yuminensis* and *F*. *walujewii* Regel, respectively, in the Flora of China (FOC, http://foc.eflora.cn/).

The morphological traits of *Fritillaria* species, particularly the *Fritillaria cirrhosa* D.Don complex (referring to *F*. *cirrhosa* and closely-related species in morphology), which are widely distributed in southwest China [2], are complex due to the high variability of several characters, including leaf width; leaf curling; petals tessellated or not, and bract number. However, the mechanism of the variation is not clear and the current classification of some species is only temporary. More comprehensive studies into the morphological variation in the genus are required to facilitate a precise and reasonable species classification [2]. Furthermore, the species occurring in Xinjiang also exhibit significant morphological variation due to the diversity of microclimates (mountains, swamps, saline conditions, and other habitats). Currently, 16 variants are recorded in Flora Xinjiangensis, though they are treated as synonyms of the corresponding accepted species names in the FOC and The Plant List (www.theplantlist.org). Certain character variations of some individuals are prominent and beyond the characteristic range of the genus, such as 8–12 petals, 4–8 stamens, and a 3–5-lobed stigma. Moreover, the stigma of most *Fritillaria* species is 3-lobed, but in a few species, i.e., *F*. *yuminensis* and *F*. *karelinii* (Fisch. ex D.Don) Baker, it is undivided. It has been proposed that an undivided stigma is a primitive characteristic [3], but physiological and molecular evidence is required to test this hypothesis and to assess the evolution of this trait within the genus.

The bulbs of some *Fritillaria* species, including *F*. *thunbergii* Miq., *F*. *cirrhosa*, *F*. *walujewii*, and *F*. *pallidiflora* Schrenk, have long been used in traditional Chinese medicine [4]. As a result, long-term excessive harvesting has led to substantial declines in the size of wild *Fritillaria* populations. At present, all of the eight species in Xinjiang have been classified as vulnerable according to the list of rare endangered endemic higher plants of Xinjiang [5], which has attracted scientific interest. The genetic diversity of some species in the genus was previously assessed, and corresponding conservation areas were proposed [6, 7]; however, some species with very narrow distributions and greater extinction threat require evaluation. A scientific approach to conservation requires an accurate understanding of the population genetic diversity and structure. The diversity estimated by different markers, such as plastid DNA, genomic inter-simple sequence repeats (ISSRs), and single nucleotide polymorphisms (SNPs), can be used to comprehensively inform conservation strategies.

The classification of the genus was previously revised where it was subdivided into eight subgenera, including *Davidii*, *Liliorhiza*, *Japonica*, *Fritillaria*, *Rhinopetalum*, *Petilium*, *Theresia*, and *Korolkowia* [8]. A later phylogenetic analysis of 37 *Fritillaria* species using *matK*, *trnK* intron, *rp116* intron, and nrDNA *ITS* [1] supported this subgeneric classification [8]. Khourang et al. investigated the phylogenetic position of nine species in Iran using the *ITS* and *trnL*-F regions [9], and showed that members of the subgenera *Fritillaria* and *Rhinopetalum* formed one clade. However, a phylogenetic study of 92 species using *matK*, *rbcL*, and *rpl16* [10] indicated that, in contrast to the results of [1, 9], *Fritillaria* appeared to be polyphyletic. Additionally, the monophyly of seven out of the eight newly classified subgenera by Rix [8] (*F*. subgenus *davidii*, *Liliorhiza*, *Japonica*, *Rhinopetalum*, *Petilium*, *Theresia*, and *Korolkowia*) was well supported. The largest subgenus (*F*. subgenus *Fritillaria*) formed two strongly supported clades, with one clade comprising taxa that occur mainly in Europe, the Middle East, and North Africa, and the other clade comprising taxa occurring in China and Central Asia [10]. However, the relationships of some of these species were not well resolved, particularly *F*. *thunbergii* Miq. and *F*. *cirrhosa*. The phylogenetic position of the Xinjiang-endemic species *F*. *yuminensis* remains unclear.

The chloroplast (cp) genome in angiosperms is highly conserved, with a quadripartite structure consisting of a large single copy (LSC) region, a small single copy (SSC) region, and two copies of a larger inverted repeat (IR). The gene orders in these regions are also similar; however, structural rearrangements and gene losses can be found in some lineages [11, 12]. Plastid sequences have been widely used for deciphering phylogenetic relationships and in DNA barcoding to identify plant species [13]. However, DNA barcoding for species identification and phylogenetic analysis is hampered by weak resolution in some plants [14–16]. Complete cp genomes have therefore emerged as a means of improving the resolution of phylogenies that have varied among, or been unresolved in, earlier single- and multi-gene studies [17–20]. With the rapid development of next-generation sequencing techniques, it is now more convenient and relatively inexpensive to obtain cp genome sequences and extend gene-based phylogenetics to phylogenomics.

To date, a total of six *Fritillaria* cp genomes have been sequenced and are available on GenBank. Park et al. reported the cp genomes of *F*. *ussuriensis* and *F*. *cirrhosa* and performed a comparative analysis with four *Fritillaria* cp genomes available on GenBank, the outcome of which has provided a basic understanding of the cp genome characteristics of the genus [21]. In the present study, we sequenced the cp genomes of seven *Fritillaria* species from Xinjiang using the Illumina HiSeq platform. The aims of this study were to (1) analyze the global structural patterns of the seven cp genomes and compare them with the six cp genomes available on GenBank; (2) discover highly divergent DNA markers that can be used for population genetics; and (3) evaluate the phylogenetic relationships of the Xinjiang species, particularly the position of *F*. *yuminensis*, and assess the evolution of an undivided stigma in the genus.

## Materials and methods

### Plant materials

Fresh leaves of seven *Fritillaria* species were collected from Tacheng and Yili Prefecture of Xinjiang Uygur Autonomous Region, China. The geographic origin and coordinates of sampling locations were listed in S1 Table. The sample collection was approved by the Forestry Bureau of Tacheng Prefecture and Yili Prefecture. For each species, two to five individuals were sampled. Voucher specimens were deposited at the Xinjiang Institute of Ecology and Geography, Chinese Academy of Sciences (S1 Table).

### Genome sequencing

Total DNA was extracted from approximately 100 mg of fresh leaves using the CTAB method following Yao et al. [22]. Illumina paired-end libraries were constructed and sequenced by the Illumina HiSeq X-Ten platform (Illumina Inc., USA) at the Germplasm Bank of Wild Species in Southwest China, Kunming Institution of Botany, Chinese Academy of Sciences. Each individual of each species was sequenced independently. In total, 22 individuals of seven species were sequenced. Because the cp genome sequences of repeat individuals of each species were almost identical, therefore, we reported only one genome of each species.

### Genome assembly and annotation

The raw reads were trimmed and assembled into contigs using SPAdes [23]. Contigs representing the cp genome were obtained after a BLAST search using the cp genome sequence of *F*. *cirrhosa* (GenBank No. KY646167) as a reference sequence. The resulting contigs were assembled after being aligned to the reference genome using Geneious 4.8 [24] and annotated using the Dual Organellar GenoMe Annotator (DOGMA) database [25]. The cp genome map was generated using OGDRAW (http://ogdraw.mpimp-golm.mpg.de/) [26]. The raw sequencing data were deposited in GenBank SRA database (SAMN08348372–SAMN08348378, https://submit.ncbi.nlm.nih.gov/subs/sra). The annotated seven cp genomes were deposited in GenBank (accession number MG200070, MG211818-MG211823).

### Genome comparison

A comparative plot consisting of full alignments of the cp genomes with annotations was produced by mVISTA using *F*. *cirrhosa* as the reference. The sequences were aligned using MEGA 6 [27] and then manually adjusted using BioEdit software (http://www.mbio.ncsu.edu/bioedit/bioedit.html). Subsequently, a sliding window analysis was conducted to evaluate the nucleotide diversity (Pi) of the cp genome using DnaSP 5.1 [28]. The step size and window length was set to 200 bp and 600 bp, respectively. The number of variable sites and the Pi across the complete cp genomes, LSC, SSC, and IR regions were calculated using DnaSP 5.1. The *p*-distance among species was calculated in MEGA 6 to evaluate the divergence of *Fritillaria* species.

### Phylogenetic analyses

Sequences of the 13 *Fritillaria* species and three *Lilium* species were aligned using MEGA 6. Phylogenies were constructed by maximum likelihood (ML) and Bayesian Inference (BI) analyses using the protein-coding genes (PCGs), LSC, SSC, and IR regions. ML analyses were conducted in MEGA 6, while BI analyses were conducted using BEAST 1.7 [29]. GTR+G+I and GTR+G were selected as the best substitution models for the ML and BI analyses according to the Akaike information criterion (AIC) [30] and Bayesian information criterion (BIC) [31], respectively, and were estimated using MrModeltest 2.3 [32]. For ML, initial tree(s) for the heuristic search were obtained automatically by applying the Neighbor-Join and BioNJ algorithms to a matrix of pairwise distances estimated using the Maximum Composite Likelihood approach. The tree was drawn to scale, with branch lengths measured in the number of substitutions per site. All alignment positions containing more than 5% gaps were eliminated. For BI, two independent Markov Chain Monte Carlo chains were conducted simultaneously for 2 × 10^7^ generations and sampled every 1,000 generations. Each run was assessed using Tracer 1.6 [33] to evaluate whether a sufficient effective sample size (ESS) had been reached. The two runs were considered as converged when the ESS of all relevant parameters was above 200. A consensus maximum clade credibility (MCC) tree was generated from the 75% post-burn-in trees using TreeAnnotator 1.7.

## Results

### Genome sequencing, assembly, and genome features

Illumina sequencing generated 3.5 to 7.1 Gb of raw reads and 164,351 to 697,002 paired-end reads for the seven *Fritillaria* species. After the *de novo* assembly, eight to 19 contigs covering the whole chloroplast genome were used to generate a complete cp genome (S1 Table). Using reference-guided assembly, seven *Fritillaria* cp genomes were obtained, with coverage of 162× to 688× for each species.

The full-length cp genomes of the seven species ranged from 151,764 in *F*. *meleagroides* Patrin ex Schult. & Schult.f. to 152,112 bp in *F*. *karelinii* (Table 1). The cp genome presented a typical quadripartite structure including one LSC region (81,533–81,879 bp), one SSC region (17,277–17,526 bp), and a pair of IR regions (52,654–52,778 bp; 26,327–26,389 each).

**Table 1:** The chloroplast genomic characteristics of 13 *Fritillaria* species.

Species	Genome (bp)	LSC (bp)	SSC (bp)	IRs (bp)	PCG	tRNA	rRNA	GC (%)	GenBank accession No.
*F*. *pallidiflora* Schrenk	152,078	81,787	17,513	26,389	78	30	4	37	MG211822
*F*. *tortifolia* X.Z.Duan & X.J.Zheng	152,005	81,778	17,509	26,359	78	30	4	37	MG211819
*F*. *walujewii* Regel	151,920	81,743	17,523	26,327	78	30	4	36.9	MG211820
*F*. *verticillata* Willd.	151,959	81,730	17,509	26,360	78	30	4	36.9	MG211823
*F*. *karelinii* (Fisch. ex D.Don) Baker	152,112	81,879	17,473	26,381	78	30	4	36.9	MG211818
*F*. *meleagroides* Patrin ex Schult. & Schult.f.	151,764	81,833	17,277	26,327	78	30	4	36.9	MG211821
*F*. *yuminensis* X.Z.Duan	151,813	81,533	17,526	26,377	78	30	4	36.9	MG200070
*F*. *ussuriensis* Maxim.	151,524	81,732	17,114	26,339	78	30	4	36.95	KY646166
*F*. *cirrhosa* D.Don	151,083	81,390	17,537	26,078	78	30	4	36.96	KY646167
*F*. *hupenesis* P.K.Hsiao & K.C.Hsia	152,145	81,898	17,553	26,347	77	30	4	37	NC024736
*F*. *taipaiensis* P.Y.Li	151,693	81,390	17,550	26,352	78	30	4	37	NC023247
*F*. *unibracteata* P.K.Hsiao & K.C.Hsia	151,009	81,290	17,541	26,089	78	30	4	37	KF769142
*F*. *thunbergii* Miq.	152,155	81,890	17,565	26,350	78	30	4	37	KY646165

**Fig 1:**
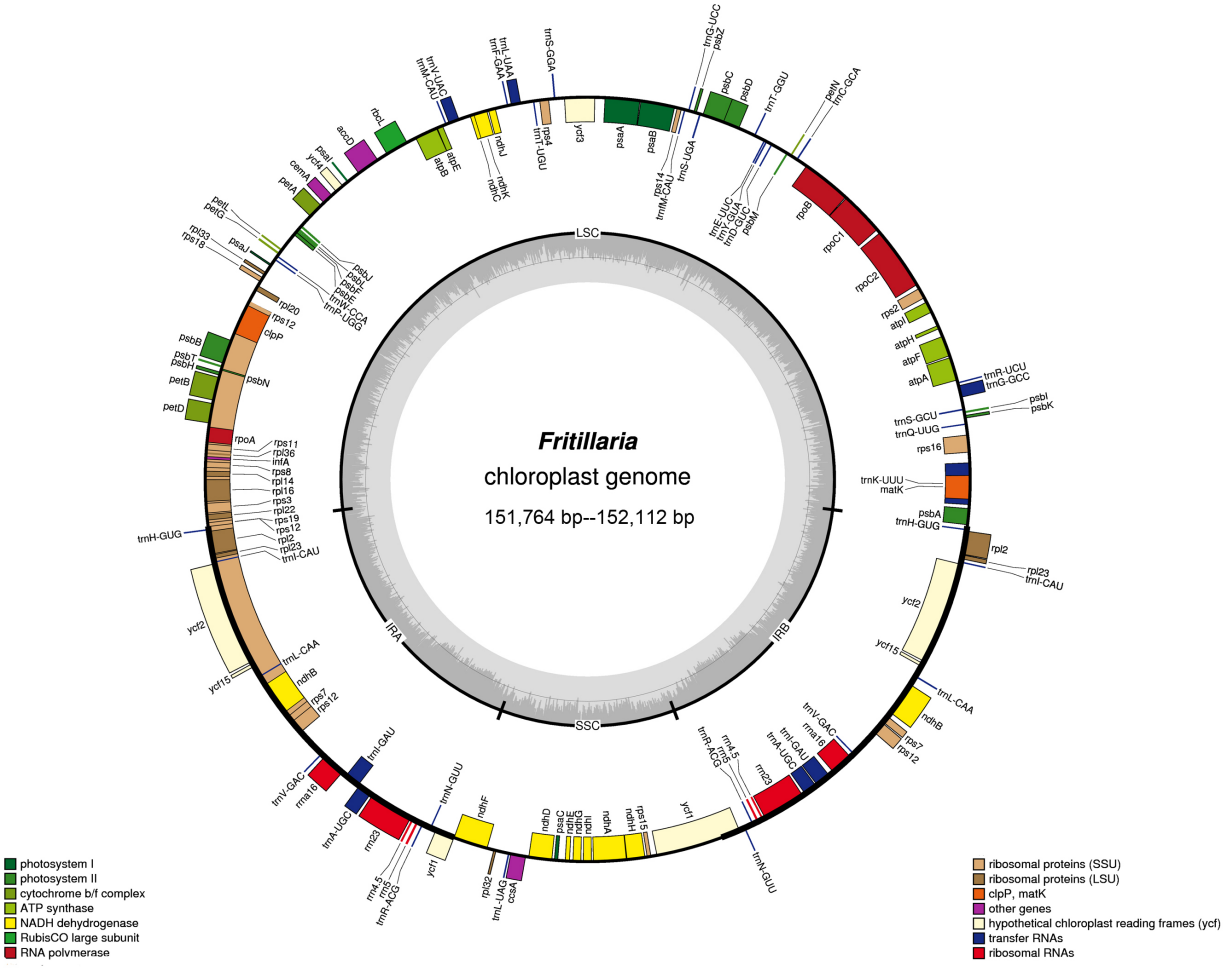
Gene map of the *Fritillaria* cp genome. Genes belonging to different functional groups are color-coded. The dashed area in the inner circle indicates the GC content. Cp genome size ranges are provided for the seven Xinjiang *Fritillaria* species.

The gene content and order were identical in the seven species. A total of 114 distinct genes were annotated, including 78 PCGs, 30 tRNA genes, four rRNA genes, *infA* (translation initiation factor gene), and hypothetical ORF *ycf15* (S2 Table). Eighteen genes were duplicated in the cp genome, including eight tRNAs (*trnA*-*UGC*, *trnI*-*CAU*, *trnI*-*GAU*, *trnL*-*CAA*, *trnN*-*GUU*, *trnR*-*ACG*, *trnV*-*GAC*, *trnH*-*GUG*), four rRNAs (*rrn16*, *rrn23*, *rrn4*.5, *rrn5*), and six PCGs (*ndhB*, *rpl2*, *rpl23*, *rps12*, *ycf2*, *rps7*). Gene *rps12* was trans-spliced because the 5′end was located in the LSC region and the 3′ end in the IR region. Gene *ycf1* in the junction region between SSC and IRb was the only pseudogene found due to the incomplete duplication of the normal copy in the junction region (Fig 1). There were 18 intron-containing genes, among which two genes (*ycf3* and *clpP*) had two introns each, while the other 16 had one intron, including 10 PCGs (*atpF*, *rpoC1*, *rpl2*, *ndhB*, *ndhA*, *petB*, *petD*, *rpl16*, *rps16*, *rps12*) and six tRNA genes (*trnA-UGC*, *trnG-GCC*, *trnI-GAU*, *trnK-UUU*, *trnL-UAA*, *trnV-UAC*) (Fig 1).

The Pi of the seven species was 0.00648. SSC had the highest Pi value, while IR had the lowest value (Table 2). The mean *p*-distance among the seven species was 0.00558, ranging from 0.003 to 0.01. The distance between *F*. *karelinii* /*F*. *meleagroides* and the other five species was larger than that between the five species (S3 Table), indicating that *F*. *karelinii* and *F*. *meleagroides* were most divergent.

**Table 2:** Variable site analyses in *Fritillaria* chloroplast genomes.

	Among 13 *Fritillaria* species			Among seven Xinjiang species		Among other six species	
Region	Total sites	Variable sites	Pi	Variable sites	Pi	Variable sites	Pi
LSC	84,114	2,227	0.00737	1,734	0.00782	1,368	0.00638
SSC	17,854	672	0.01044	518	0.0112	346	0.00774
IR	26,550	152	0.00148	126	0.00169	59	0.00094
Complete cp genome	154,837	3,199	0.00557	2,498	0.00684	1,625	0.00419

### Genome sequence divergence

We compared the Pi of the LSC, SSC, and IR regions of the cp genome. In total, 3,199 variable sites were found (Pi = 0.00557), indicating moderate genetic divergence of the *Fritillaria* cp genomes. The IR regions exhibited the lowest Pi (0.00148), while SSC had the highest Pi (0.01044) (Table 2). The *p*-distances among the *Fritillaria* species ranged from 0.0001 to 0.01, and *F*. *karelinii*, *F*. *meleagroides*, and *F*. *ussuriensis* exhibited the greatest sequence divergence. The Pi of the Xinjiang species (0.00648) was higher than that of the species from the other regions (0.00419), as the two highly divergent species *F*. *karelinii* and *F*. *meleagroides* are from Xinjiang.

### Junction characteristics

**Fig 2:**
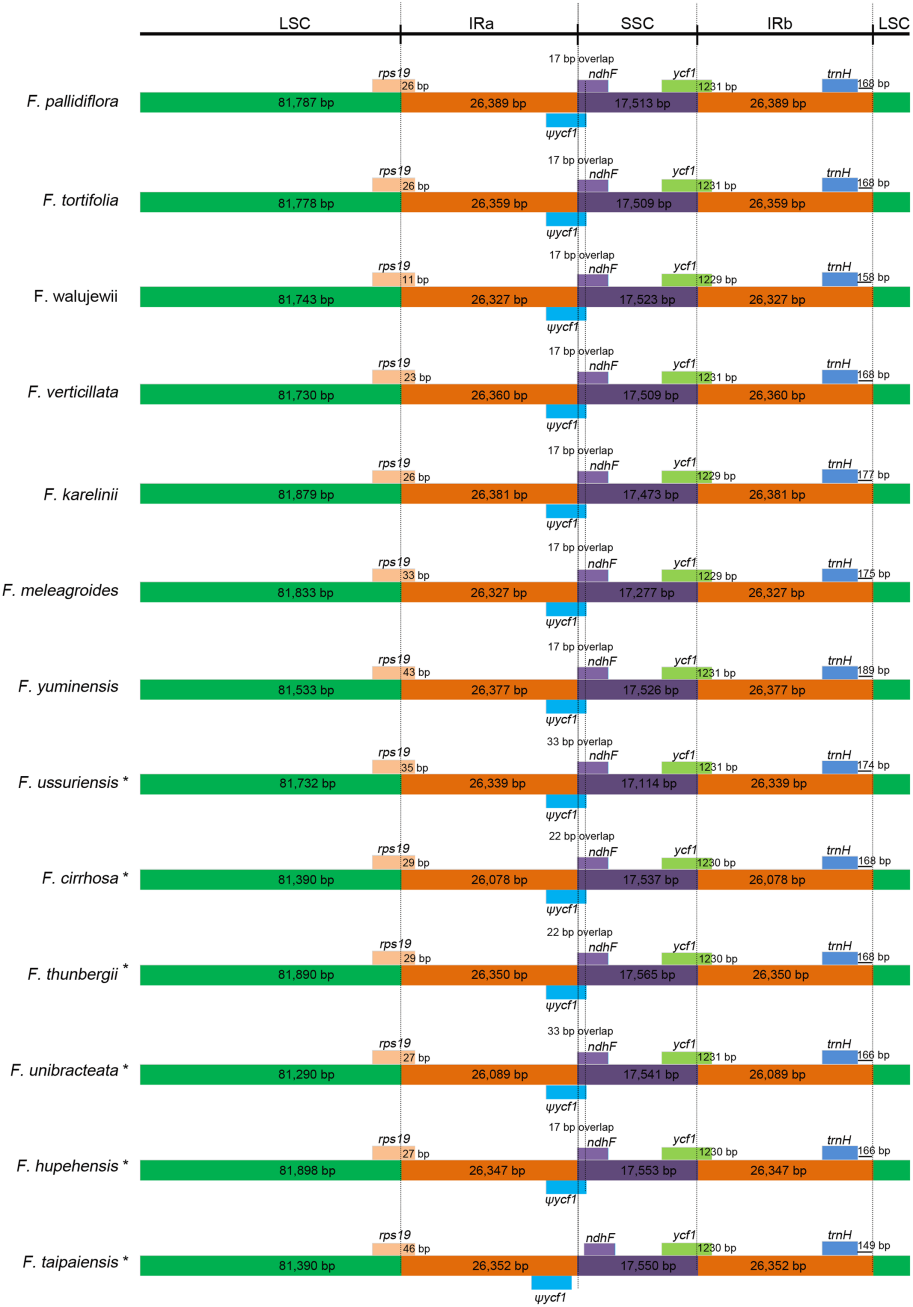
Comparison of LSC, SSC, and IR border regions among the 13 *Fritillaria* cp genomes. Colored boxes for genes represent the gene position. ψ: pseudogenes. *: these six species were redrawn according to Park et al. [21].

The junction of the LSC, SSC, and IR regions of the seven species are shown in Fig 2. The *rps19* gene located in the LSC was extended into the IRa by 11–43 bp. The border between IRa/SSC and SSC/IRb extended into the *ycf1* genes. Overlaps of 17 bp were found between the *ycf1* pseudogene and the *ndhF* gene. The *trnH* genes were all located in the IR region, 158–189 bp from the IRb/LSC boundary.

### Genome-wide comparative analyses

**Fig 3:**
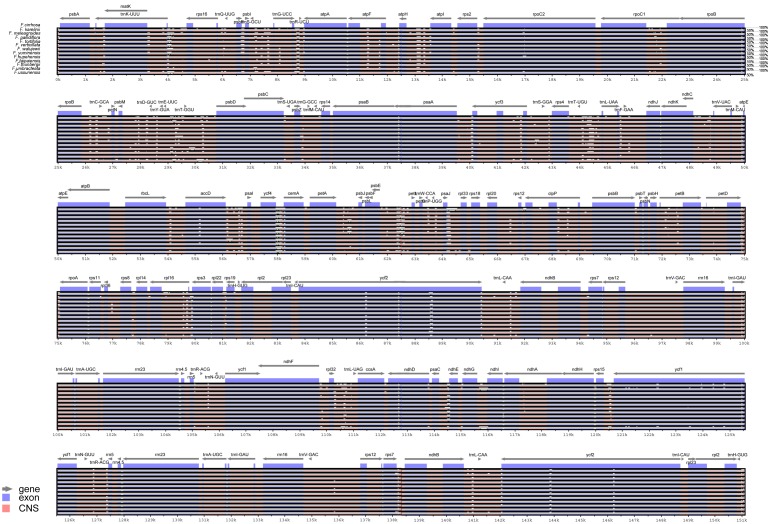
Comparison of 13 *Fritillaria* cp genomes with *F*. *cirrhosa* as the reference. LSC: long single copy region; SSC: short single copy region; IRa and IRb: inverted regions. Gray arrow: gene and translation direction; blue block: exon of the gene; red block: conserved non-coding sequences (CNS). Sequence identities are labeled at the right side and range between 50%-100%.

**Fig 4:**
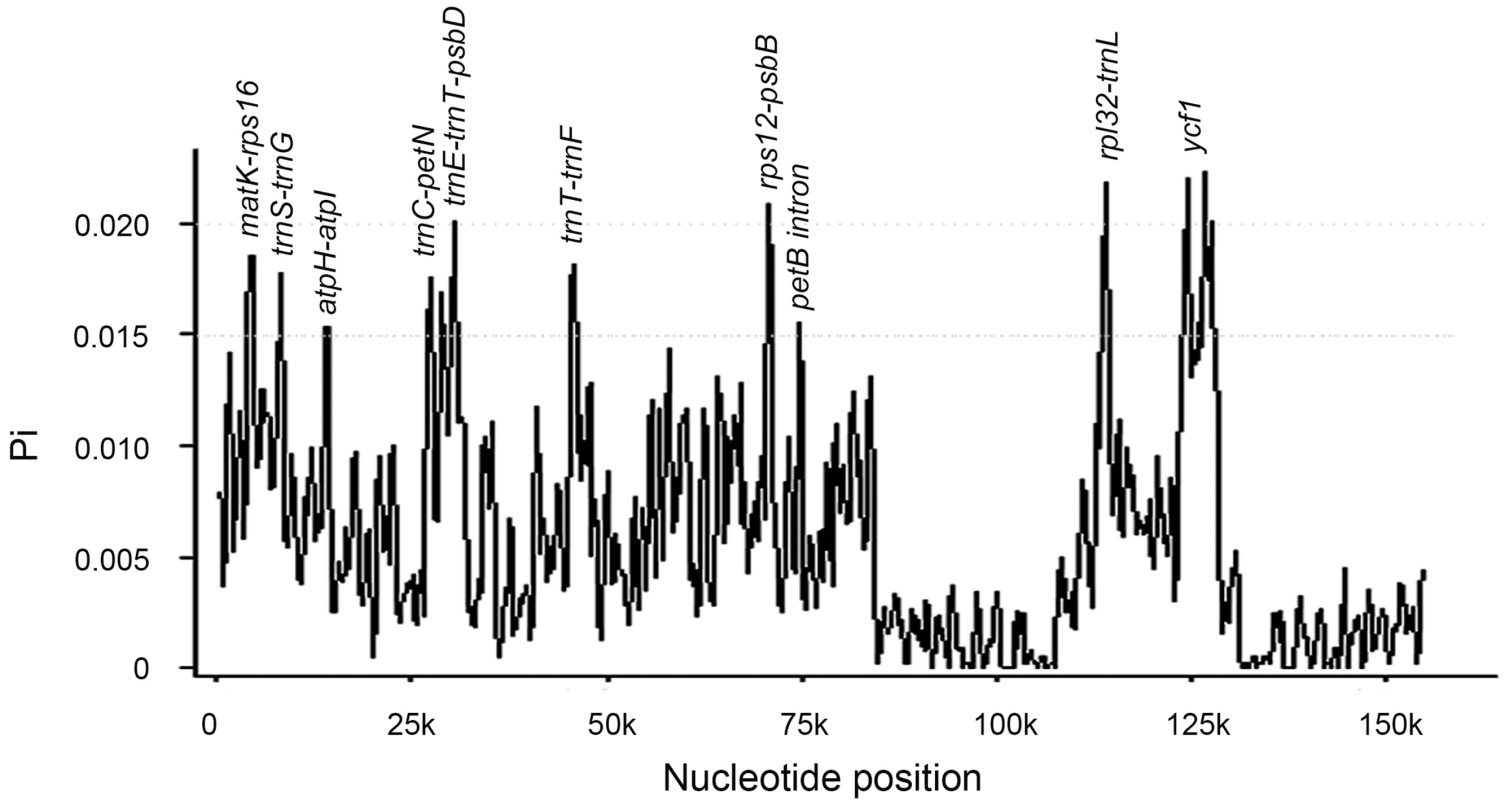
Sliding window analysis of the entire chloroplast genome of 13 *Fritillaria* species.

We aligned the 13 *Fritillaria* cp genomes using mVISTA, and found that the gene order and clusters were very similar in all the species (Fig 3). Using sliding window analysis, we identified the 10 most divergent regions that could be utilized as potential molecular markers for population genetic and phylogenetic studies in *Fritillaria*. These regions included *matK-rps16*, *trnS-trnG*, *atpH-atpI*, *trnC-petN*, *trnE-trnT-psbT*, *trnT-trnL-trnF*, *rps12-psbB*, *rpl32-trnL* in IGS, and the *petB* intron and *ycf1* in the coding region (Fig 4). Additionally, *psbB-psbH*, *petD-rpoA*, *ycf4-cemA*, and *ycf2* also constitute potential candidates.

### Phylogenetic analyses

The phylogenetic analyses were conducted with the PCGs, LSC, SSC, and IR regions using ML and BI inference methods. All the analyses revealed congruent tree topologies, and all branches were highly supported (S1 Fig). Two clades were identified among the 13 *Fritillaria* species. Clade I contained *F*. *ussuriensis*, *F*. *meleagroides*, and *F*. *karelinii*. The sister clade (clade II) comprised the remaining 10 species, in which two subclades were strongly supported. Subclade I contained species from South China (*F*. *cirrhosa*, *F*. *unibracteata*, *F*. *taipaiensis*, *F*. *hupehensis*, and *F*. *thunbergii*). Subclade II included five Xinjiang species, namely *F*. *tortifolia*, *F*. *verticillata*, *F*. *yuminensis*, *F*. *pallidiflora*, and *F*. *walujewii*. The seven Xinjiang species did not form a monophyletic group, as *F*. *meleagroides* and *F*. *karelinii* were separated from the other five species. Additionally, *F*. *yuminensis* had a close phylogenetic relationship with *F*. *tortifolia* and *F*. *verticillata*.

## Discussion

In this study, seven new cp genomes of *Fritillaria* were sequenced and ranged in size from 151,764 to 152,112 bp. The reported *Fritillaria* cp genome size in this study is consistent with previously sequenced *Fritillaria* cp genomes, and is also within the cp genome size range of angiosperms. The gene content and gene order were the same in the seven Xinjiang species, containing 78 PCGs, 30 tRNA genes, four rRNA genes, and the *infA* and *ycf15* genes. Compared with the other six species, the tRNA and rRNA genes were identical, but the PCGs differed, ranging from 77 to 78 due to the absence of the *clpP* gene in the cp genome of *F*. *hupehensis*. The hypothetical gene *ycf68* was present in the cp genome of *F*. *unibracteata*, while *ycf15* was absent from *F*. *taipaiensis*, *F*. *thunbergii*, and *F*. *ussuriensis*. The absence or presence of some genes in a particular species of a genus has also been observed in *Ipomoea* [34]. The functions of *ycf15* and *ycf68* are ambiguous in various land plants; for instance, *ycf15* in *Ipomoea purpurea* and *Ageratina adenophora* encode a complete RF15 protein, but the former has no *ycf68*, while the latter has one incomplete *ycf68* open reading frame. In *Musa acuminata*, these two genes were determined as non-functional due to the presence of several stop codons in the gene sequence [34].

The boundaries between IR and LSC or SSC were identical, except in *F*. *taipaiensis*. The LSC/IRa boundary of *Fritillaria* is located in the *rps19* gene, and a small section of the 5′end of *rps19* is in the IRb region, which is similar to *Ilex* [22] and Brassicaceae species [35–38]. In contrast, *rps19* does not extend into the IR in *Lupinus luteus* [39] and *Millettia pinnata* [40], while in others, such as *Phaseolus vulgaris* [41] and *Oryza* [42], the whole gene is contained inside the IR. ψ*ycf1* spans the SSC/IRa boundary and overlaps with the *ndhF* gene in most of the *Fritillaria* species. However, these are separated and located at each side of the boundary in the *F*. *taipaiensis* cp genome, which has also been observed in *Petroselinum crispum* (HM596073), *Tiedemania filiformis* (HM596071), and *Panax ginseng* (AY582139). The SSC/IRb boundary is inside the *ycf1* gene, which is consistent with many plants, including those from Asteraceae [43], *Ilex* [22], *Lilium* [44], and *Ananas* [45]. Conversely, in *Cryptochloa strictiflora* [46] and *Ipomoea batata* [34], the junction falls into the *ndhF* gene due to the loss of the *ycf1* gene. The *trnH* gene is duplicated in the IRs in *Fritillaria*, as observed in *Lilium* [44], whereas *trnH* is a single cope gene located in the LSC of other species, such as *Ipomoea batata* [34], *Datura stramonium* [47], and *Citrus aurantiifolia* [48].

The genomic structure and gene order of the *Fritillaria* cp genomes are highly conserved, and no rearrangement has occurred. The IRs of the *Fritillaria* species were about 26 kb, which is within the size range of most angiosperm cp genomes (20–28 kb). The IR usually varies between 200 and 300 nucleotides in seed plants. However, the extreme expansion of the IRs has been observed in *Oenothera* (54 kb) [49], Fabaceae (50 kb) [50], and *Pelargonium*×*hortorum* (75 kb) [51]. In contrast, the loss or near loss of the IR has also been also detected in *Erodium* and *Sarcocaulon* [52]. These significant contractions and expansions of the IR contribute towards genome size variation.

Several variable cp DNA markers have been used in phylogenetic studies of *Fritillaria*, for instance *rbcL*, *matK*, and *atpB*. Some divergent intergenic spacers, i.e., *trnH-psbA*, *rpl32-trnL*, *psbB-psbH*, and *trnS-trnG*, are more informative and suitable in lower taxonomic ranks [53]. Upon comparison of the 13 cp genomes, the 10 most divergent regions were identified, and included *matK-rps16*, *trnS-trnG*, *atpH-atpI*, *trnC-petN*, *trnE-trnT-psbT*, *trnT-trnL-trnF*, *rps12-psbB*, and *rpl32-trnL* in IGS, and the *petB* intron and *ycf1* in the coding region. The Pi of these regions ranged from 0.015 to 0.022. Additionally, *psbB-psbH*, *petD-rpoA*, *ycf4-cemA*, and *ycf2* also constitute potential candidates, which corroborates previous studies [44]. These highly divergent regions (also called hotspots) in the cp genome are useful for further phylogenetic and population genetics studies. However, in contrast to Park et al. [21], we found the *petB* intron to be highly divergent. Furthermore, the *clpP* intron was also found to be highly variable, as reported in *Acacia ligulata* [54]. Gene *ycf1* is considered as the most promising plastid DNA barcode of land plants [55].

Universal DNA barcoding is widely used in the identification of plant species, but has several limitations [14–16]. The complete cp genome, as a super DNA barcode, has been successfully used in numerous phylogenetic studies of seed plants [56, 57] and in resolving species relationships at lower taxonomic levels [58]. Park et al. conducted a phylogenetic study of six *Fritillaria* species based on the cp genome and concluded that plastome phylogenies are suitable for uncovering relationships among *Fritillaria* species, and obtain good support with high bootstrap values [21]. We constructed phylogenetic trees of 13 *Fritillaria* species using the PCG, LSC, SSC, and IR datasets. The phylogenetic relationships within the genus were identical and strongly supported in all of the phylogenies. In this study, *Fritillaria* appears to be a monophyletic group, which differs from the results of Day et al. [10] and may be attributed to our smaller sampling size. However, the positions of *F*. *cirrhosa* and *F*. *thunbergii* are far more highly resolved in our study (S1 Fig).

**Fig 5:**
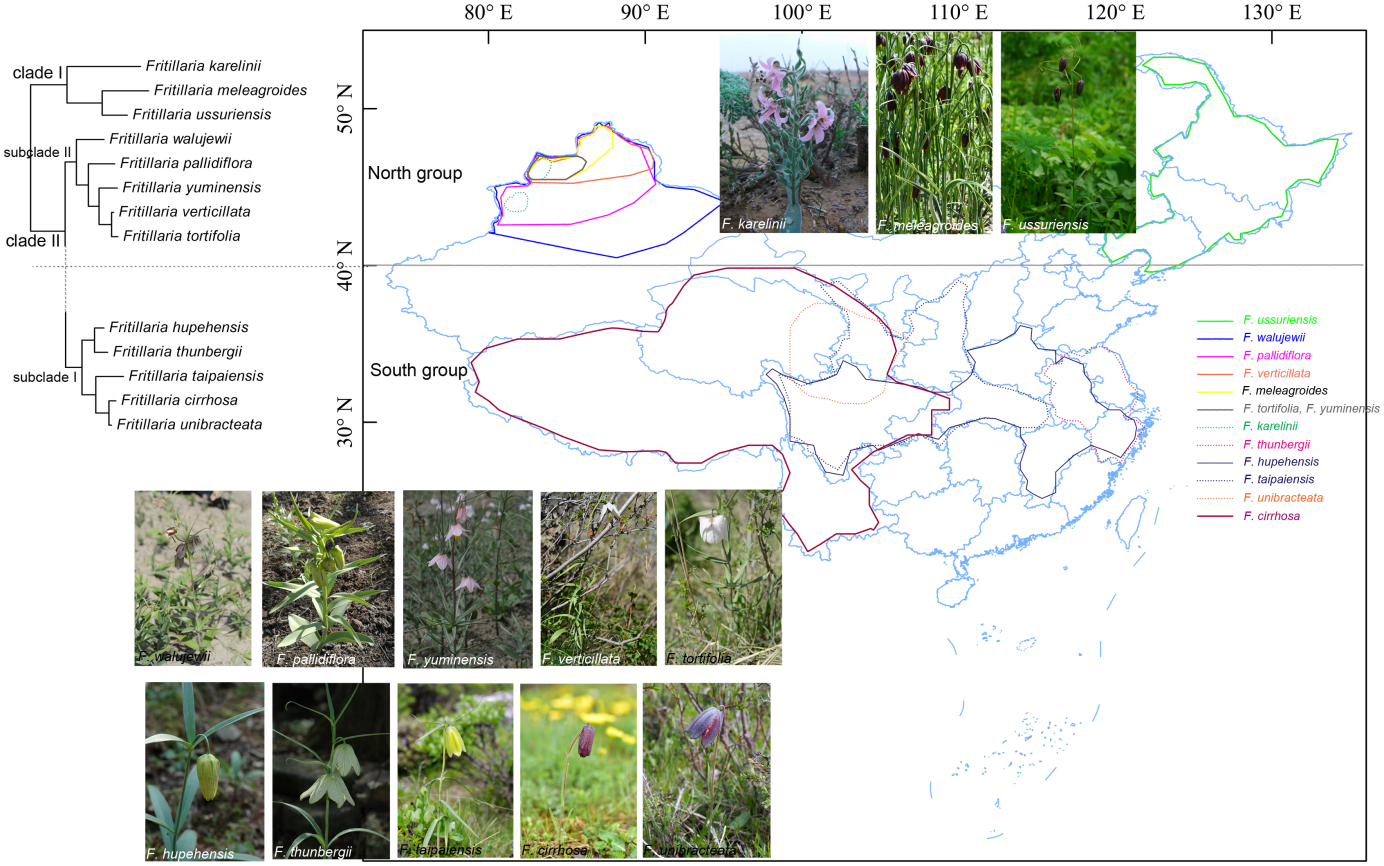
Map indicating the distribution of *Fritillaria* in China. The distribution area of each species is drawn according to the records in the FOC and Flora Xinjiangensis. Photographs of the species are also provided.

**Fig 6:**
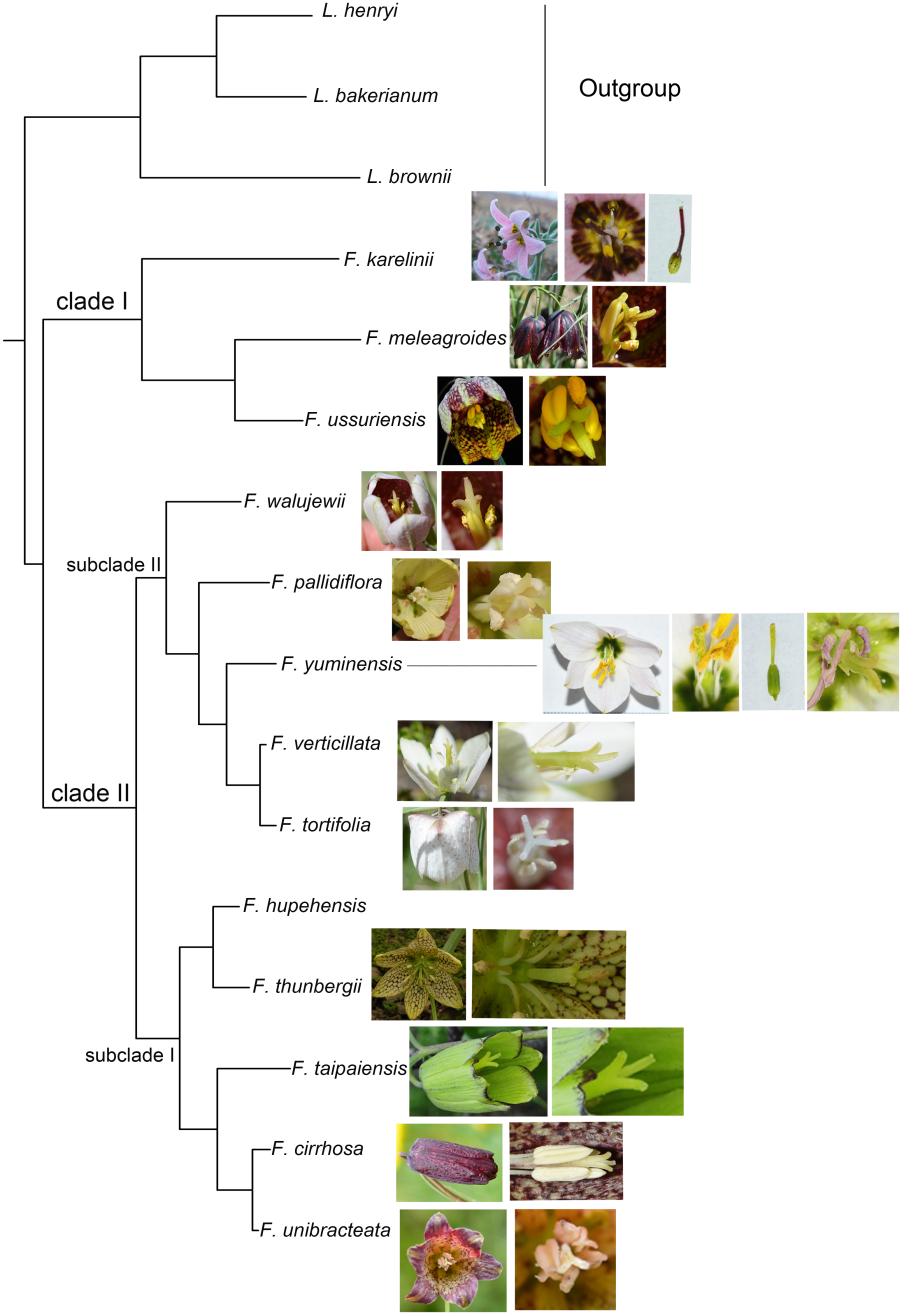
The evolutionary progression of stigmatic traits within the genus.

With some exceptions, our phylogenies are largely consistent with Day et al. [10] and support the polyphyletic classification of *F*. subgenus *Fritillaria* by Rix [8]. Two species of *F*. subgenus *Fritillaria* (*F*. *meleagroides* and *F*. *ussuriensis*) clustered together and are sister to *F*. *karelinii* of *F*. subgenus *Rhinopetalum* in clade I (Figs 5 and 6), which is similar to the results of Khourang et al. [9], and may be attributed to the small sample size. The other 10 species of *F*. subgenus *Fritillaria* formed a strongly supported clade (clade II), and two subclades were resolved in clade II. The five species from outside Xinjiang formed a strongly supported subclade (subclade I), which was sister to subclade II containing the other five Xinjiang species. This indicated that the Xinjiang species had a close genetic affinity. Interestingly, we found that the eight species in clade I and subclade II of clade II originate from Xinjiang and Heilongjiang in North China (named the “north group”), and the five species in subclade I of clade II originate from South China (named the “south group”). An alternative explanation of the phylogenetic pattern is that the southern taxa diverged from the northern taxa and become distinct due to limited seed flow or genetic contact.

The seven Xinjiang species did not form a monophyletic group, as *F*. *karelinii* and *F*. *meleagroides* were highly divergent from the other five species, and, interestingly, also differ in their morphology and habitat. Specifically, *F*. *meleagroides* occurs in a variety of habitats, including hilly slopes, shallow waters in mountainous areas, saline areas, and shallow swamps, while *F*. *karelinii* can usually be found in the plains of *Artemisia* desert habitats (desert habitat dominated by some drought tolerant *Artemisia* species) or low gravel hills. This species has a style that is longer than the stamens, and the stigma is scarcely lobed and slightly inflated at the top (Figs 5 and 6).

Endemic species are often limited to specific geographic areas, and in many instances have evolved vicariantly [59]. Previous studies have demonstrated that specific limiting factors in an environment can significantly influence the geographic distribution patterns of species, including physical factors (i.e., temperature, light, moisture, aridity) and biotic factors (i.e., competition, predation, food availability). These factors usually influence the survival and propagation ability of plants. For instance, *Corynephorus canescens* is widely distributed in mid and south Europe, and its northern distribution limit in Europe coincides with the 15°C isotherm in July, as its germination and flowering are affected by low temperature [60]. The winter distribution and abundance patterns of several avian species are directly linked to their physiological limits, with the northern range limit being associated with the −4°C isotherm of the average minimum January temperature [61]. As high solar radiation and temperature are most favorable for the C4 photosynthetic pathway, C4 grass abundance patterns in North America are separated at 40°N, where the C4 grass abundance is above 50% north of 40°N and below 50% south of 40°N [62].

Interestingly, we also discovered that the northern and southern groups were largely separated at 40°N (Fig 5). However, the determining factor(s) influencing the distribution of *Fritillaria* species are not investigated in the present study. However, we hypothesize that soil moisture is an important environmental constraint influencing the growth of *Fritillaria* and other spring ephemeral plants. A semi-arid or desert climate prevails in Xinjiang and the precipitation is very low. Adequate water supply is only available from snow melting during March to June. From late June, the climate turns dry and hot, and is not suitable for growth. They have therefore adapted to a complete growth cycle ahead of the hot summer. Conversely, in south China, such as Sichuan, Hubei, and Zhejiang, precipitation is greater in summer, and thus some species have much longer growth cycles and can thrive from August to October (i.e., *F*. *cirrhosa*) (S4 Table).

The stigma in the majority of *Fritillaria* species is 3-lobed; however, in a few species, i.e., *F*. *yuminensis* and *F*. *karelinii*, the stigma is undivided (Fig 6). We surveyed 48 species in FOC and Flora of USSR, and found that only four species possess a scarcely lobed stigma. It was proposed that the trait of an undivided stigma might be a primitive characteristic [3]. Our results do not support this hypothesis. The phylogenies demonstrate that *F*. *karelinii* diverges early, while *F*. *yuminensis* does not, and *F*. *karelinii* is closely related to *F*. *tortifolia* and *F*. *verticillata*. Moreover, in comparison to the phylogeny of Day et al. [10], *F*. *karelinii* is not resolved as a basal species. Therefore, at this stage, we cannot infer a definite evolutionary trend for this trait. More cp genomes need to be sequenced to gain a comprehensive and accurate assessment of the evolutionary progression of the stigma. Furthermore, as *F*. *yuminensis* and *F*. *karelinii* do not form a monophyletic clade, this suggests that this trait might have evolved independently several times in the genus.

Additionally, wild *Fritillaria* populations have been dramatically reduced due to excessive harvesting in recent decades. During our field investigation, we noted that *F*. *meleagroides* and *F*. *karelinii* were rare in the wild. The endemic species *F*. *yuminensis* is now endangered and can only be found in remote areas that are uninhabited by humans and livestock. Small populations of the other endemic species *F*. *tortifolia* can only be found in remote areas and natural reserves. Although all seven species in Xinjiang are listed in the class I protection plant list of Xinjiang, conservation action is urgently required. Population diversity is an important index in the formulation of a scientific conservation strategy. The newly sequenced cp genomes of these seven *Fritillaria* species would be useful for the development of SSR markers, and together with the identified divergent regions DNA regions, could be used to comprehensively assess the genetic diversity of wild populations in order to inform the protection of these valuable medicinal resources.

As there are more than 140 species in the genus, the currently sequenced species only represent a very limited sample. However, we provide evidence that the cp genome can increase the resolution of phylogenetic relationships within the genus. More cp genomes are required to clarify the taxonomic and phylogenetic relationships of *Fritillaria* species at lower taxonomic levels, and can be used to estimate the population genetic diversity in order to formulate effective protection strategies.

## Supporting information

S1 Table Sampled species and their voucher specimens. (DOCX)

S2 Table Genes present in the seven *Fritillaria* cp genomes. (DOCX)

S3 Table Paired genetic distance between seven *Fritillaria* species in Xinjiang. (DOCX)

S4 Table The distribution areas and habitats of the 13 *Fritillaria* species in China. (DOCX)

S1 Fig Phylogenetic relationships of the 13 *Fritillaria* species inferred from ML and BI analyses using different data partitions. (A) PCGs, (B) LSC region, (C) SSC region, and (D) IR region. Values above the branches represent ML bootstrap values/BI posterior probability values. Outgroup: *Lilium brownii* KY748296; *L*. *bakerianum* KY748301; *L*. *henryi* KY748302.
(TIF)
