# Supplementary material for: Complete chloroplast genome of seven Fritillaria species, variable DNA markers identification and phylogenetic relationships within the genus
Source: PLoS One. 2018 Mar 15;13(3):e0194613. doi: 10.1371/journal.pone.0194613 (PMC5854438; doi:10.1371/journal.pone.0194613)
Supplement: S1 Table — (DOCX) [file pone.0194613.s001.docx]

**S1 Table. Sampled species and their voucher specimens used in this study**

| **Species** | **geographic origin** | **geographic coordinates** | **voucher specimens** | **mapped reads** | **raw data** | **Contig No.** | **Coverage (×)** |
| --- | --- | --- | --- | --- | --- | --- | --- |
| *F. pallidiflora* | Huocheng county, Xinjiang | 44°30′N, 80°48′E | WY01622 | 336,519 | 3.8G | 11 | 332 |
| *F. tortifolia* | Baerluke, Yumin county, Xinjiang | 82°30′N, 45°54′E | WY01619 | 336,433 | 3.9G | 11 | 332 |
| *F. walujewii* | Sailimu, Yili, Xinjiang | 44°30′N, 81°05′E | WY01625 | 697,002 | 7.1G | 19 | 688 |
| *F. verticillata* | Duolate, Tuoli county, Xinjiang | 45°55′N, 83°40′E | WY01620 | 556,627 | 5.8G | 16 | 549 |
| *F. karelinii* | Yumin county, Xinjiang | 46°28′N, 82°48′E | WY01626 | 680,628 | 4.9G | 8 | 671 |
| *F. meleagroides* | Tuoli county, Xinjiang | 45°56'N, 83°37'E | WY01624 | 655,221 | 5.4G | 8 | 648 |
| *F. yuminensis* | Tacheng, Xinjiang | 46°45′N, 82°57′E | WY01623 | 164,351 | 3.5G | 9 | 162 |
