# Supplementary material for: Complete chloroplast genome of seven Fritillaria species, variable DNA markers identification and phylogenetic relationships within the genus
Source: PLoS One. 2018 Mar 15;13(3):e0194613. doi: 10.1371/journal.pone.0194613 (PMC5854438; doi:10.1371/journal.pone.0194613)
Supplement: S2 Table — (DOCX) [file pone.0194613.s002.docx]

**S2 Table. Genes present in the seven *Fritillaria* chloroplast genome**

| **Gene group** | **Gene name** |
| --- | --- |
| Transfer RNAs | *trnA-UGC**, *trnC-GCA*, *trnD-GUC*, *trnE-UUC*, *trnF-GAA*, *trnG-UCC*, *trnG-GCC*, *trnH-GUG**,*trnI-CAU**, *trnI-GAU**, *trnK-UUU*, *trnL-UAA*, *trnL-UAG*, *trnL-CAA**, *trnM-CAU*, *trnfM-CAU*, *trnN-GUU**, *trnP-UGG*, *trnQ-UUG*, *trnR-ACG**, *trnR-UCU*, *trnS-GCU*, *trnS-GGA*, *trnS-UGA*, *trnT-GGU*, *trnT-UGU*, *trnV-UAC*, *trnV-GAC**, *trnW-CCA*, *trnY-GUA* |
| Ribosomal RNAs | *rrn16**, *rrn 23**, *rrn 4.5** , *rrn 5** |
| Ribosomal protein small subunit | *rps2*, *rps* 3, *rps 4*, *rps 7**, *rps 8*, *rps 11*, *rps 12** , *rps 14*, *rps 15*, *rps 16*, *rps 18*, *rps 19* |
| Ribosomal protein  Large subunit | *rpl2** , *rpl14*, *rpl16* , *rpl20*, *rpl22*, *rpl23**, *rpl32*, *rpl33*, *rpl36* |
| Subunits of RNA polymerase | *rpoA*, *rpoB*, *rpoC1* , *rpoC2* |
| photosystem I | *psaA*, *psaB*, *psaC*, *psaI*, *psaJ* |
| Photosystem II | *psbA*, *psbB*, *psbC*, *psbD*, *psbE*, *psbF*, *psbH*, *psbI*, *psbJ*, *psbK*, *psbL*, *psbM*, *psbN*, *psbT*, *psbZ* |
| Cythochrome b/f complex | *petA*, *petB* , *petD* , *petG*, *petL*, *petN* |
| ATP synthase | *atpA*, *atpB*, *atpE*, *atpF* , *atpH*, *atpI* |
| NADH-dehydrogenase | *ndhA* , *ndhB** , *ndhC*, *ndhD*, *ndhE*, *ndhF*, *ndhG*, *ndhH*, *ndhI*, *ndhJ*, *ndhK* |
| Large subunit Rubisco | *rbcL* |
| Other | *infA*, *accD*, *ccsA*, *matK*, *clpP*, *cemA* |
| Conserved hypothetical chloroplast ORF | *ycf1**, *ycf 2**, *ycf 3* , *ycf 4*, *ycf 15** |

*: indicating gene has two copies.
