# Supplementary material for: Complete chloroplast genome of seven Fritillaria species, variable DNA markers identification and phylogenetic relationships within the genus
Source: PLoS One. 2018 Mar 15;13(3):e0194613. doi: 10.1371/journal.pone.0194613 (PMC5854438; doi:10.1371/journal.pone.0194613)
Supplement: S3 Table — (DOCX) [file pone.0194613.s003.docx]

**S3 Table. Paired genetic distance between seven *Fritillaria* species in Xinjiang.**

|  | *F. pallidiflora* | *F. tortifolia* | *F. walujewii* | *F. verticillata* | *F. karelinii* | *F. meleagroides* | *F. yuminensis* |
| --- | --- | --- | --- | --- | --- | --- | --- |
| *F. pallidiflora* |  |  |  |  |  |  |  |
| *F. tortifolia* | 0.003 |  |  |  |  |  |  |
| *F. walujewii* | 0.003 | 0.004 |  |  |  |  |  |
| *F. verticillata* | 0.003 | 0.001 | 0.004 |  |  |  |  |
| *F. karelinii* | 0.01 | 0.01 | 0.01 | 0.01 |  |  |  |
| *F. meleagroides* | 0.009 | 0.01 | 0.009 | 0.009 | 0.008 |  |  |
| *F. yuminensis* | 0.003 | 0.002 | 0.004 | 0.002 | 0.01 | 0.01 |  |
