# Supplementary material for: Complete chloroplast genome of seven Fritillaria species, variable DNA markers identification and phylogenetic relationships within the genus
Source: PLoS One. 2018 Mar 15;13(3):e0194613. doi: 10.1371/journal.pone.0194613 (PMC5854438; doi:10.1371/journal.pone.0194613)
Supplement: S4 Table — (DOCX) [file pone.0194613.s004.docx]

**S4 Table. The distribution areas and habitats of the 13 *Fritillaria* species in China.**

| **Species** | **Fistribution areas** | **Habitat** | **Altitude (m)** | **Flowering (fl.) and fruiting (fr.) time** |
| --- | --- | --- | --- | --- |
| *F. pallidiflora* | NW Xinjiang | forests, thickets, meadows, grassy slopes, mountain steppes | 1300-2500 | fl. May, fr. Jun. |
| *F. tortifolia* | NW Xinjiang | thickets, alpine grassy slopes | 1500-2100 | fl. Apr-May, fr. Jun. |
| *F. walujewii* | Xinjiang | openings in *Picea* forests, thickets, meadows, steppes | 1300-2000 | fl. May,fr. Jun |
| *F. verticillata* | NW Xinjiang | hill thickets, gravelly meadows | 1300-2000 | fl. Apr-Jun, fr. Jul |
| *F. karelinii* | NW Xinjiang | sandy soil, stony slopes, gravelly screes |  | fl. Apr-May, fr. Jun |
| *F.meleagroides* | NW Xinjiang | mud flats, wet meadows, swampy fields | 900-2400 | fl. May-Jun |
| *F. yuminensis* | NW Xinjiang | forest margins, open gravelly slopes | 1700-2800 | fl. Apr, fr. May-Jun |
| *F. ussuriensis* | Heilongjiang, Jilin, Liaoning | forests, thickets, meadows, streamsides, shady and moist places | below 500 | Fl. May-Jun, fr. Jul |
| *F. cirrhosa* | Gansu, Qinghai, Sichuan, Xizang, Yunnan | forests, alpine thickets, meadows, flood lands, moist places | 3200-4600 | fl. May-Jul, fr. Aug-Oct |
| *F.unibracteata* | S Gansu, SE Qinghai, NW Sichuan | thickets, meadows | 3200-4500 | fl. May-Jun, fr. Aug |
| *F. taipaiensis* | Gansu, Hubei, Shaanxi, Sichuan | hill thickets, grassy slopes | 2000-3200 | fl. May-Jun, fr. Jun-Jul |
| *F. hupehensis* | Anhui, Henan, Hubei,Jiangxi, Sichuan, Zhejiang. | forests, moist places on limestone hills, flood lands | 100-1600 | fl. Apr-Jun, fr. Jun-Jul |
| *F. thunbergii* | Anhui, Jiangsu, Zhejiang | bamboo forests, shady and moist places | below 600 | fl. Mar-Apr, fr. May--Jun |
