# Supplementary figures and images for: Complete chloroplast genome of seven Fritillaria species, variable DNA markers identification and phylogenetic relationships within the genus
Source: PLoS One. 2018 Mar 15;13(3):e0194613. doi: 10.1371/journal.pone.0194613 (PMC5854438; doi:10.1371/journal.pone.0194613)

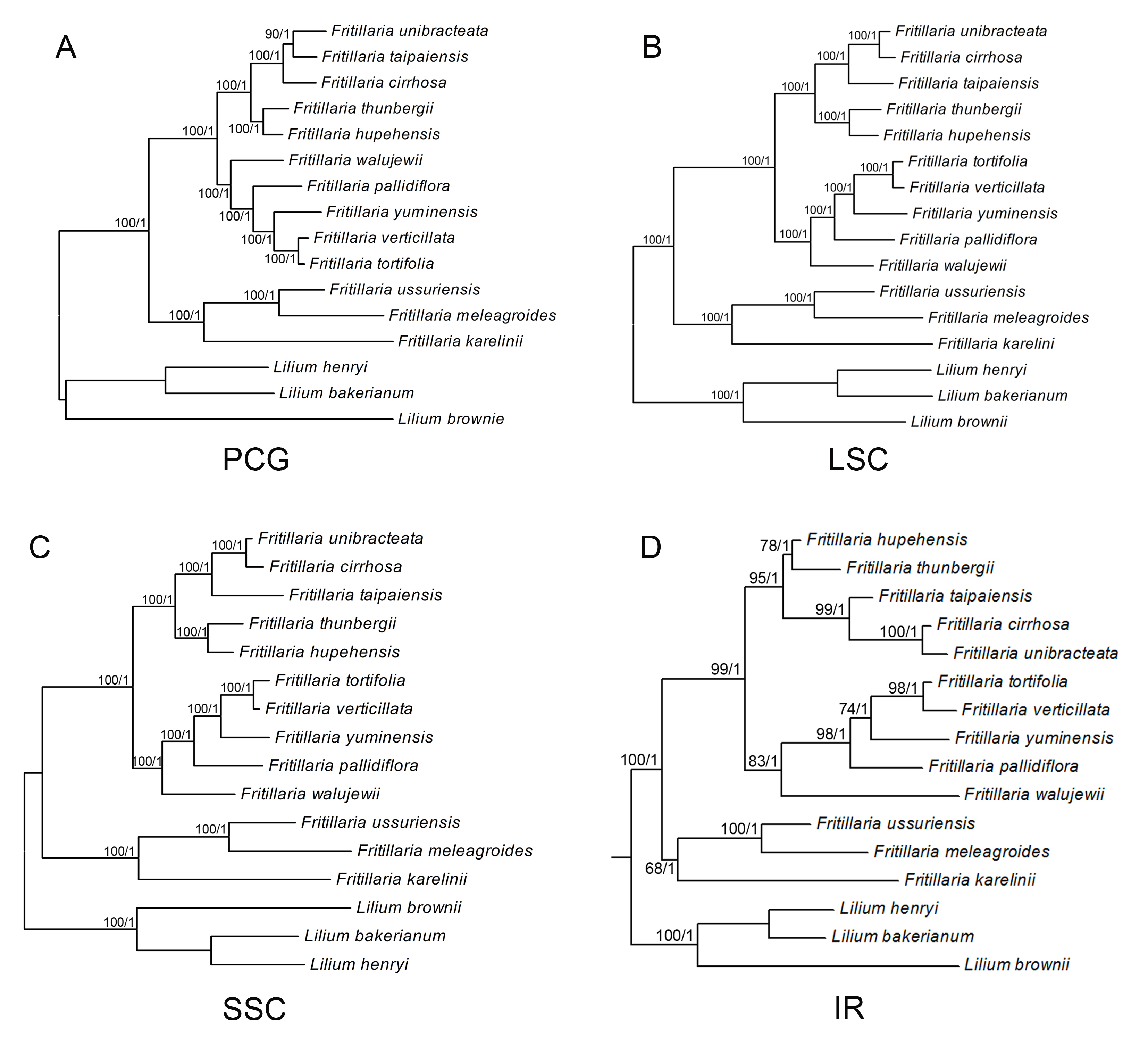

Supplement: S1 Fig — (A) PCGs, (B) LSC region, (C) SSC region, and (D) IR region. Values above the branches represent ML bootstrap values/BI posterior probability values. Outgroup: Lilium brownii KY748296; L. bakerianum KY748301; L. henryi KY748302. (TIF) [file pone.0194613.s005.tif]
